# Visuoauditory Associative Memory Established with Cholecystokinin Under Anesthesia Is Retrieved in Behavioral Contexts

**DOI:** 10.1523/JNEUROSCI.1673-19.2019

**Published:** 2020-03-04

**Authors:** Zicong Zhang, Xuejiao Zheng, Wenjian Sun, Yujie Peng, Yiping Guo, Danyi Lu, Yilin Zheng, Xiao Li, Peter Jendrichovsky, Peng Tang, Ling He, Min Li, Qing Liu, Fuqiang Xu, Gabriel Ng, Xi Chen, Jufang He

**Affiliations:** ^1^Departments of Biomedical Sciences, Biology, and Chemistry, City University of Hong Kong, Hong Kong, China,; ^2^Department of Rehabilitation Sciences, The Hong Kong Polytechnic University, Hong Kong, China,; ^3^City University of Hong Kong Shenzhen Research Institute, Shenzhen, Guangzhou, China,; ^4^Wuhan Institute of Physics and Mathematics, Chinese Academy of Sciences, Wuhan, China 430071, and; ^5^Guangzhou Institute of Biomedicine and Health, Chinese Academy of Sciences, Guangzhou, China 510530

**Keywords:** auditory cortex, cholecystokinin, entorhinal cortex, memory encoding, neural plasticity, operant conditioning

## Abstract

Plastic change in neuronal connectivity is the foundation of memory encoding. It is not clear whether the changes during anesthesia can alter subsequent behavior. Here, we demonstrated that in male rodents under anesthesia, a visual stimulus (VS) was associated with electrical stimulation of the auditory cortex or natural auditory stimulus in the presence of cholecystokinin (CCK), which guided the animals' behavior in a two-choice auditory task. Auditory neurons became responsive to the VS after the pairings. Moreover, high-frequency stimulation of axon terminals of entorhinal CCK neurons in the auditory cortex enabled LTP of the visual response in the auditory cortex. Such pairing during anesthesia also generated VS-induced freezing in an auditory fear conditioning task. Finally, we verified that direct inputs from the entorhinal CCK neurons and the visual cortex enabled the above neural plasticity in the auditory cortex. Our findings suggest that CCK-enabled visuoauditory association during anesthesia can be translated to the subsequent behavior action.

**SIGNIFICANCE STATEMENT** Our study provides strong evidence for the hypothesis that cholecystokinin plays an essential role in the formation of cross-modal associative memory. Moreover, we demonstrated that an entorhinal–neocortical circuit underlies such neural plasticity, which will be helpful to understand the mechanisms of memory formation and retrieval in the brain.

## Introduction

The hippocampal system consists of the hippocampus and adjacent entorhinal, perirhinal, and parahippocampal cortices (Squire and Zola-Morgan, 1991). The entorhinal and perirhinal cortices are the gateway between the hippocampus and the neocortex and have strong reciprocal connections with the entire neocortex (Swanson and Köhler, 1986; Canto et al., 2008). Observations that patients with hippocampal system damage show difficulties forming new long-term memories for facts and events (Scoville and Milner, 1957; Corkin, 1984) led to our understanding that the hippocampal system is essential for establishing long-term memories (Squire and Zola-Morgan, 1991). However, patients with hippocampal damage can still recall remote memories (Teng and Squire, 1999; Wang et al., 2009; Lesburguères et al., 2011), suggesting that the neocortex stores these memories (Graham and Hodges, 1997; Squire et al., 2001). Previously, we found the establishment of long-term visuoauditory associative memory by pairing a visual stimulus (VS) with electrical stimulation in the auditory cortex of rats was critically dependent on the entorhinal cortex (Chen et al., 2013).

Many neurons in the entorhinal cortex contain cholecystokinin (CCK) (Innis et al., 1979; Greenwood et al., 1981; Köhler and Chan-Palay, 1982), and the release of CCK is controlled by NMDA receptors (Chen et al., 2019). The released CCK of entorhinocortical projections in the auditory cortex produces long-term potentiation (LTP) and sound–sound associative memory (Chen et al., 2019). They also enable the visuoauditory association in the auditory cortex by responding to a formerly ineffective sound or light stimulus after pairing with a noise-burst stimulus (Li et al., 2014). Infusion of a CCK antagonist into the auditory cortex prevents the formation of this visuoauditory association, similar to inactivation of the entorhinal cortex (Chen et al., 2013; Li et al., 2014). Conscious recall of intraoperative events under general anesthesia is rare (Kihlstrom et al., 1990; Ghoneim and Block, 1997), though there are cases where this occurs when the anesthesia is light (Lubke et al., 2000).

To test our hypothesis that CCK is a memory-writing chemical for visuoauditory associative memory in the neocortex, we investigated whether the presence of CCK allows the formation of associative memory in the cortex of anesthetized rats that is retrievable in a behaviorally relevant context. In anesthetized rats, electrical stimulation in the auditory cortex (EAC) or acoustic stimulus (AS) was paired with a visual stimulus (VS) in the presence of CCK. The rats were subsequently tested if they could use the VS as an associative cue to retrieve a water reward in a two-choice task. We then examined how neurons respond to the associated VS and what neuron types were involved in this visuoauditory association using optogenetics. We also investigated whether stimulating the axon terminals of CCK neurons in the AC could induce CCK-mediated visuoauditory associations. Finally, we investigated whether the convergence of entorhinal and visual projections in the AC accounts for this visuoauditory association.

## Materials and Methods

### 

#### 

##### Animals.

Male Sprague Dawley rats (8 weeks old) with clean external ears were used for the behavioral study with no surgery and for pre-experimental sessions in which they were trained to use two tones as cues for reward retrieval. Rats were surgically implanted with electrodes and cannulae, and those that successfully finished the behavioral experiments were included in the study. Both male and female (8 to 10 weeks old) C57/BL/6 (C57, wild-type), *CCK-ires-Cre* (Cck^tm1.1(Cre)Zjh/J^, C57 background), and CCK-CreER (Cck^tm2.1(Cre/ERT2)Zjh/J^, C57 background, CCK^−/−^) mice were used for immunohistochemistry, *in vivo* extracellular recordings, and behavioral experiments. Animals were confirmed to have clean external ears and normal hearing and were housed in a 12 h light/12 h dark cycle. All procedures were approved by the Animal Subjects Ethics Subcommittees of City University of Hong Kong and the Hong Kong Polytechnic University.

##### Auditory and visual stimuli.

The AS was digitally generated using a computer-controlled Tucker-Davis Technologies Auditory Workstation and delivered through a coupled electrostatic speaker (EC1, Tucker-Davis Technologies). The sound pressure level was calibrated with a condenser microphone (Center Technology). Pure tones of 60–70 dB SPL were used to screen rats in the pretraining experiments. The VS was white light generated by light-emitting diodes placed 5 cm above the center hole of the behavioral apparatus. When the light was on, the illumination at the bottom of the apparatus was 26 Lux.

##### Implantation surgery for electrophysiology and drug infusion.

Rats were anesthetized with sodium pentobarbital (50 mg/kg, i.p.; Ceva Sante Animale), and anesthesia was maintained at a dose of 15 mg/kg/h. Atropine sulfate (0.05 mg/kg, s.c.). was administered 15 min before anesthesia to inhibit tracheal secretions. A local anesthetic (xylocaine, 2%) was liberally applied to the incision site. Animals were prepared for surgery, as previously described (Chen et al., 2013). Rats were mounted in a stereotaxic device, and a midline incision was made in the scalp after a liberal application of a local anesthetic (2% xylocaine). Bilateral craniotomies were performed over the temporal lobe (3.0–6.5 mm posterior, 3.0–5.0 mm ventral to bregma) to access the AC, and the dura matter was opened. Body temperature was maintained at 37–38°C with a heating blanket.

Before electrode implantation, tungsten microelectrodes with impedances of 1–3 MΩ (Frederick Haer) were used to identify the AC. Electrodes were positioned with an oil hydraulic micromanipulator controlled from outside the soundproof room. Neuronal signals recorded by the microelectrode, together with auditory and visual signals, were amplified and stored using Tucker-Davis Technologies OpenEX and Molecular Devices Axoscope software.

We then implanted guide cannulae for drug infusion and homemade electrode arrays, which typically consisted of six electrodes (one stimulating electrode, 4 recording electrodes, and one reference electrode) into the AC of each hemisphere. Stimulating electrodes were made of insulated stainless-steel wire (A-M Systems) with an impedance of <100 kΩ. Reference and recording electrodes were made of insulated tungsten wire (California Fine Wire) with an impedance of 0.5–1 MΩ. The electrode array was held by a micromanipulator and penetrated the cortex. Electrodes in the two hemispheres were deliberately implanted symmetrically at slightly different locations to avoid invoking strong commissural connections. Ground electrodes for stimulation and recording were separately connected to screws on the skull. The electrodes were advanced to a depth of 1000–1100 μm where neurons showed stable auditory responses, and the skull opening was covered with a layer of silicone (World Precision Instruments). The connection sockets of the electrodes were cemented to the skull with the cannulas. Rats were then housed in their home cages and recovered for 5 d before experiments.

For experiments in mice, CCK-ires-Cre and CCK^−/−^ mice were anesthetized with pentobarbital sodium (0.8 mg/kg, i.p.), and anesthesia was maintained throughout surgery and neuronal recordings with periodic supplements. Atropine sulfate (0.05 mg/kg, s.c.) was administered 15 min before the induction of anesthesia to inhibit tracheal secretions. Briefly, animals were mounted in a stereotaxic device, and a midline incision was made in the scalp. A craniotomy was performed at the temporal lobe (2–4 mm posterior, 1.5–3 mm ventral to bregma) to access the AC, and the dura mater was opened. In the experiment in which virus injection was applied to the entorhinal cortex, another craniotomy was performed (4–5 mm posterior, and 2.5–3.5 mm lateral to bregma).

##### AC stimulation and reward retrieval.

Five days after surgery, rats underwent behavioral training, during which they learned to retrieve a water reward cued by perceiving EAC. Water was restricted to 50% of normal intake before training. Rats were placed in a homemade cage with 3 horizontally aligned holes with infrared sensors and were first manually guided to poke their noses into the center hole before moving to the left or right hole, where a drop of water (15–20 μl) was delivered. Rats then underwent formal training consisting of 4 stages. In stage 1, a nose-poke in the center hole triggered a high-frequency (HF) or low-frequency (LF) sound stimulus, which indicated specific hole for reward retrieval (i.e., for 7 rats, HF or LF indicated the left or right hole, respectively; vice versa for the other rats; see Movie 1). In stage 2, rats were required to perceive EACL or EACR (5 pulses at 80–150 μA, 20 Hz) delivered after a nose-poke in the center hole. Pulses were generated by the Tucker-Davis Technologies system and delivered through the stimulating electrode (of low impedance) via an isolator (ISO-Flex; A.M.P.). Stimulation of the left AC indicated reward availability in the right hole, and stimulation of the right AC indicated reward availability in the left hole. Infrared sensors detected whether rats arrived at the correct hole within 5 s from the onset of stimulation, in which case a drop of water was delivered. Stimulations were delivered to only one hemisphere in one session (100 trials) and switched to another hemisphere in another session until a 0.9 correct rate was reached. In stage 3, electrical stimulations were delivered to one hemisphere to another hemisphere for 10 trials each. Rats were required to reach a correct rate of 0.9 or higher. In stage 4, electrical stimulations were delivered to either hemisphere in a pseudo-random manner. Training finished when rats reached a correct rate of 0.9 or higher (see Movie 2).

Movie 1. Behavioral response to AS in the two-choice water retrieval task training. 10.1523/JNEUROSCI.1673-19.2019.video.1

Movie 2. Behavioral response to EAC in the two-choice water retrieval task training. 10.1523/JNEUROSCI.1673-19.2019.video.2

##### CCK local infusion and pairing the VS with EAC during anesthesia.

After training, rats underwent a baseline testing phase during which a VS (white light, 500 ms duration, and 26-Lux illumination) was randomly presented at a very low probability (10 of 1000 trials). Behavioral responses to the light were recorded. No water reward was delivered with the presentation of the VS. Responses to 10 presentations of the VS were collected.

Rats were then anesthetized with ketamine/xylazine. AC neuronal responses to the VS were measured before cholecystokinin octapeptide (CCK-8, 0.5–1.0 μl, 10 ng/μl, 0.1 μl/min, Tocris Bioscience) was infused into the left or right AC. Within 15 min after infusion, VS was then paired with EAC in the targeted hemisphere (2 pulses at 80–150 μA, 20 Hz). In each trial, VS (500 ms) was presented, followed by EAC at the offset of VS. Forty pairings (10 s interstimulation interval in between) occurred on each day for 3–4 d. Neuronal responses to the VS in both the targeted and naive hemispheres were recorded.

After full recovery from anesthesia, postintervention tests of behavioral responses to the VS were performed every week for up to 4 weeks. A “decision index” was assigned to quantify behavioral response. When the left hole was the target hole for the visuoauditory association, approaching the left hole was scored 1; not approaching to either hole was scored 0.5; and approaching the right hole was scored 0. When the right hole was the target hole, scores were assigned vice versa. To record neuronal activity in response to the VS, animals were placed in the training apparatus, and the VS was presented for 30 times and the corresponding neuronal activities were recorded.

##### Vehicle controls.

To eliminate the possibility of nonspecific effects of infusion or other manipulations of rats during anesthesia, we designed 2 control experiments. First, we infused CCK-8 into one hemisphere and artificial CSF (ACSF) into the other hemisphere at the same time and paired the VS with simultaneous EAC of both hemispheres. Second, we infused ACSF into one hemisphere and performed stimulus pairings. One hour later, we infused CCK-8 into other hemisphere and repeated the stimulus pairings.

##### Natural auditory stimulus and water retrieval task.

Water was restricted to 50% of normal intake before training. On day 1, rats were placed in the training cage and were first manually guided to poke their noses into the center hole before moving to the left or right hole, where a drop of water (15–20 μl) was delivered. On day 2 and 3, the rat was anesthetized with ketamine/xylazine (87 mg+13 mg/kg, i.p.). Cholecystokinin tetrapeptide (CCK-4, 24 μg/kg, 10 μl/min; Abcam) was infused through a tail-vein for 10 min, during which a visual stimulus (VS, a light flash; duration: 500 ms, 26 Lux illumination) was paired repeatedly for 60 trials with a noise-burst stimulus at one side of the box (target side) (200 ms, interstimulus interval 10 s). As the control, saline (0.9% sodium chloride) was infused in the same way for 10 min, during which the VS was paired with the presentation of the sound stimulus at the opposite side (nontarget side). The order of infusions between CCK-4 and saline was reversed for half of the subjects. The interval between the two pairings was 10 min. The time interval between CCK-4 and saline infusion is much longer than the half-life of CCK-4 in rat plasma (<1 min), which is sufficient to allow CCK-4 to be degraded before another pairing session (Koulischer et al., 1982). Starting from day 5, rats then underwent stage-2 formal training. The rats were trained to poke to the center hole to initiate a trial, then approach the left hole for reward when the sound was delivered to the left speaker outside the training box and approach the right hole for reward when the sound was delivered to the right speaker. Infrared sensors detected whether rats arrived at the correct hole within 5 s from the onset of stimulation, and a drop of water was delivered upon a correct response. Sound stimuli were delivered to the left and right speakers each for 10 trials and then each for five trials, and finally either side in a pseudorandom manner. The training was accomplished when the rat reached a correct rate of 0.85 or higher.

The rats then underwent the postintervention tests from day 9 to day 11. The same VS in the pairing session on day 2 and 3 was presented in place of sound stimulus in an occurrence of 1 in 10 trials. The behavioral responses to the VS and auditory stimuli were recorded. We defined a “decision index” to calculate the rat's response to the VS and assigned the value of “1” when the rat approached the side where the sound was paired with the VS followed by CCK-4 injection, and the value of “0” when the rat approached the opposite side.

##### Optogenetic stimulation of auditory neurons and pairing with VS in the rats.

AAV-CaMKIIa-ChR2-mCherry (1E+13 gc/ml, 1 μl, UNC vector core) was infused into the rat's primary cortex (5.0 mm posterior, 4.0 mm ventral to bregma). 4 weeks later, the animal was anesthetized with pentobarbital sodium, and the auditory neurons were confirmed by their responsiveness to the laser stimulus of 473 nm and the AS. CCK-8 was then infused into the AC, and 500 ms VS was presented for 30 trials, each of which was followed by a 5 ms laser pulse at the end. The intertrial interval was 10 s. After pairing, auditory neurons' response to VS was monitored for 10 min.

##### Quantification of neuronal responses.

Neuronal and behavioral responses were recorded simultaneously using a computer. Single-unit spikes were distinguished using spike sorting software (OpenSorter; Tucker-Davis Technologies). We considered 3 SDs above baseline as the threshold to distinguish spikes. K-means clustering method in OpenSorter was adopted to sort single-unit spikes. A unit with the largest amplitude and normal overlaid spike profile was chosen from each electrode. Another criterion was that the number of spikes with an interspike interval of <2 ms in the histogram should be <0.2% of the total number of spikes. The timing of spike occurrence relative to stimulus delivery was calculated using MATLAB software. Peristimulus time histograms (PSTHs) were calculated over a bin size of 20 ms for AC responses and 50 ms for visual responses.

Because there was no guarantee of recordings from the same unit across days, we used Z-scores (mean ± SE) to characterize neuronal responses and compare responses across different time points. *Z*-scores of neuronal responses to visual stimuli within a certain time period were calculated against the mean spontaneous firing rate within the same period, thereby representing the distance between the neuronal responses and the mean of spontaneous firing in units of SD (Z = (*x*-μ)/δ; where *x* is the neuronal response in each trial, and μ and δ are the mean and SD, respectively, of spontaneous firing rates across all trials). Higher Z-scores typically indicate a larger neuronal response, although they also differ depending on the total number of testing trials. Changes in *Z*-score after each conditioning session were used to assess the effectiveness of conditioning to induce neuronal plasticity. Paired Student's *t* tests were used to compare neuronal responses with spontaneous neuronal activity. One-way repeated-measures (RM) ANOVA was used to test for differences in mean *Z*-scores before and at different times after stimulus pairing sessions. Tukey's *post hoc* tests were used for mean comparisons. Statistical significance was set at *p* < 0.05.

##### Association between the EAC and VS in the mice.

AAV-DIO-ChR2-mCherry (1E+13 gc/ml, UNC vector core) was infused into the entorhinal cortex of CCK-ires-Cre or CCK^−/−^ mice (4.3 mm posterior, 3.2 mm lateral, and 4.5 mm ventral to bregma). Tamoxifen (150 mg/kg, i.p.) was injected for 5 consecutive days, starting from 4 d after virus injection. Mice recovered for at least 6 weeks to allow the virus to infect entorhinal cortical neurons. An optical fiber with 4 electrodes (∼200 kΩ, 300 μm between electrodes; California Fine Wire) was implanted into the AC. Neuronal responses measured by field potential and unit responses to the laser stimulation of the AC, the AS, and the VS were recorded by the implanted recording electrodes.

Single-pulse EAC was used as a conditioned stimulus in the cued fear conditioning protocol. Initially, 5 pulses (0.5 ms, 5 Hz, 100–150 μA) were applied. The onset delay between the first pulse and the foot shock (0.5 s, 0.5 mA) was 5 s. The number of pulses was gradually reduced to one based on mouse performance. After mice showed >2 s of freezing within the first 5 s of a single pulse, the VS as used before was used to test the baseline of freezing. We then anesthetized mice and recorded baseline fEPSPs in response to the VS. HF (80 Hz, 5 pulses, 5 ms duration, 10 mW) or LF (1 Hz, 5 pulses) laser stimulation was delivered to the AC, followed 1 s later by 5 pairings of the VS and single pulse EAC at 1 Hz. The onset of the VS was 100 ms earlier than the onset of EAC. This stimulation protocol was repeated 4 times with a 10 s interval. fEPSPs in response to the VS were recorded for 15 min before and after the pairing. A second pairing was performed 15 min after the first pairing, and fEPSPs were recorded for another 15 min. Neuronal responses to the VS that were 3 SDs above or below the mean were excluded. Mean fEPSP slopes before and after the pairings were calculated by linear regression and analyzed using two-way ANOVA. Freezing responses to the VS and EAC were measured 24–48 h after the pairing and analyzed using one-way ANOVA, separately.

##### Recording of fEPSP evoked by laser stimulation on the projecting terminals with opsin expression or natural sound.

To separately activate auditory cortical projections from entorhinal and visual cortices, AAV9-Syn-Flex-Chronos-GFP (3.7E+12 gc/ml, Boyden/UNC vector core) and AAV9-Syn-ChrimsonR-tdTomato (4.1E+12 gc/ml, Boyden/UNC vector core) were injected into the entorhinal cortex and visual cortex, respectively. CCK-iRES-Cre mice (The Jackson Laboratory) were adopted as the subjects. The animal was first mounted on the stereotaxic instrument (68001; RWD Life Science) followed by the sterilization of the scalp with 70% ethanol. After the midline incision, the periosteum on the skull was removed and the skull position was adjusted carefully so that bregma and lambda were on a horizontal plane, and the left and right sides of the skull were also on a horizontal plane with equal height. To approach entorhinal cortex, a 1 × 1 mm^2^ craniotomy window was made with the coordinates of −4.2 mm posterior to bregma (AP) and 3.5 mm lateral to the midline (ML) as the center, and then the injector was vertically penetrated to the depth of 3000 μm below the pia. Five mins later, 300 nl of the virus was infused at a rate of 30 nl/min. The injector stayed for 5 min after injection to allow virus spreading and avoid spilling. For the visual cortex, to avoid the spreading of the virus directly into the AC, the medial part of the visual cortex was chosen as the injection area. Two locations distributed rostral-caudally with different coordinates (AP −3.0 mm posterior to bregma, ML 1.7 mm lateral to midline; AP −4.0 mm, ML 1.7 mm) were adopted. The injector was vertically penetrated to the depth of 500 μm and then 150 nl of the virus was injected at each location.

To avoid cross talk between the two wavelengths upon laser stimulation, we used the use of two nonoverlapping lasers, 473 nm and 635 nm, to stimulate the projecting terminals in the AC from the entorhinal cortex with Chronos expression and from the visual cortex with ChrimsonR expression, respectively. The power of 473 nm laser and 635 nm laser at the optic fiber end was maintained <30 mW/mm^2^ and 40 mW/mm^2^, respectively. To avoid the photoelectric effect, fEPSPs evoked by laser stimulation were recorded by glass pipette electrodes with an impedance ∼1 MΩ rather than any other metal electrodes.

fEPSPs to 635 nm laser and noise stimuli were recorded for >15 min before pairing as the baseline, during when the 635 nm laser and noise were alternately delivered with an interval of 5 s. Then, HF (40 Hz, 5 ms, 10 pulses) or LF (1 Hz, 5 ms, 10 pulses) laser stimulation of the entorhino-cortical projection terminals in the AC followed by the pairing of the laser stimulation of visuoauditory projection terminals and the noise stimulus, which was repeated for 5 times with an intertrial interval of 10 s (abbreviated as follows: HF_ENT/VALS/noise or LF_ENT/VALS/noise), was given. After pairing, fEPSPs were recorded for another 60 min. Based on the Hebbian theory, noise was given 10 ms before 635 nm laser during pairing, considering the noise response latency of the AC. Mean fEPSP slopes before and after the pairings were normalized and calculated by linear regression and analyzed using two-way ANOVA followed by Bonferroni pairwise comparison.

##### Immunohistochemistry.

CCK-ires-Cre and CCK^−/−^ mice injected with AAV-DIO-ChR2-mCherry were fully anesthetized by an overdose of pentobarbital sodium and perfused with 30 ml of cold PBS and 30 ml of 4% paraformaldehyde. Brain tissue was removed, postfixed, and treated with 30% sucrose at 4°C for 2 d. Brain tissue was sectioned on a cryostat (40 μm) and preserved with antifreeze buffer (20% glycerin and 30% ethylene glycol diluted in PBS) at −20°C. Sections were incubated with anti-Dsred (Takara, 632496, Rabbit 1:500) multiclonal antibody at 4°C for 48 h. After 4 times PBS washing, sections were incubated with secondary antibody (Jackson Laboratories, 488, 111302) for 1.5 h at 37°C. All the sections were mounted on slides and subsequently imaged (20× magnification) by using an LSM 710 confocal microscope (Zeiss).

##### Assessment of viral expression pattern.

The extent of virus expression for rats and mice was examined by outlining the regions of expression on coronal sections from individual animal redrawn from rat or mouse brain atlas and superimposing all animals at 70% transparency to highlight the average expression for each group.

##### Quantification and statistical analysis.

All statistical analysis (including paired Student's *t* test and one, two or three-way RM ANOVA was done in Prism 7 (GraphPad Software) or SPSS. Pairwise comparisons were adjusted by Bonferroni correction. Statistical significance was set at *p* < 0.05.

## Results

### Visuoauditory association between EAC and VS with local CCK infusion in the anesthetized rat

**Figure 1.**
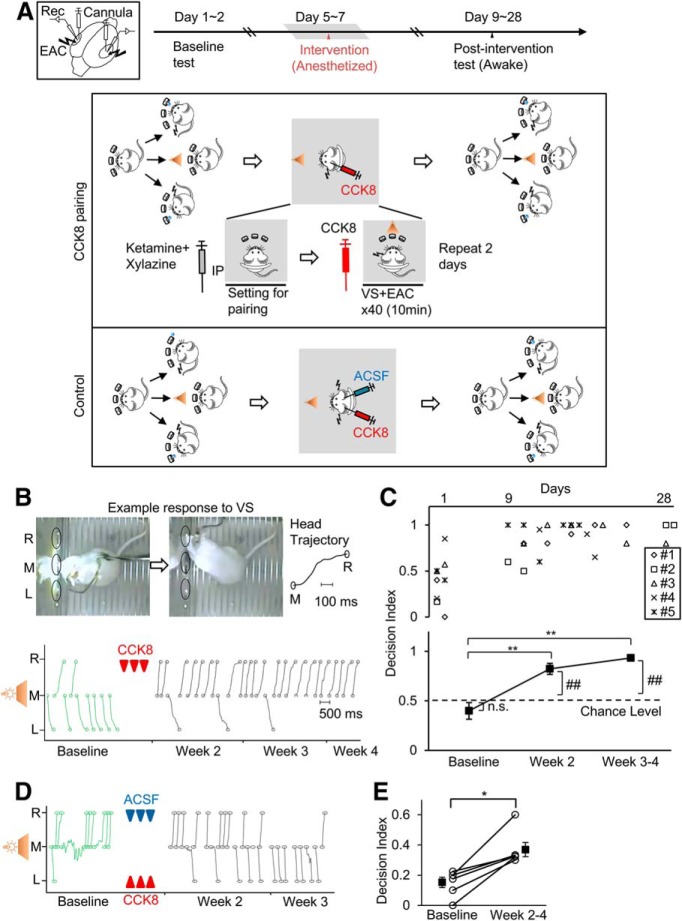
Visuoauditory association established between the VS and the auditory cortical stimulation with CCK-8 local infusion. ***A***, Schematic presentation of electrode and injection cannula placement, timeline and diagram for the experiment. Rats underwent three experimental phases after training: baseline test, CCK-8 infusion, and pairing during anesthesia and postintervention test. For vehicle control, CCK and ACSF were simultaneously infused into each hemisphere, respectively, before pairings of the VS and EAC of both hemispheres. ***B***, Behavioral responses (trajectory of head movement) to the VS during baseline (green curves) and postintervention (black curves) testing of a representative rat. L, Left hole; R, right hole; M, middle hole. Stimulus pairings with CCK infusion are marked by red arrowheads. ***C***, Individual values (top) and mean ± SEM (bottom) for decision index across weeks. **p < 0.01, one-way ANOVA with *post hoc* Dunnett's multiple-comparisons tests; ## *p* < 0.01, n.s., not significant, compared with chance level 0.5, single sample *t* tests. ***D***, Behavioral responses to the VS from a representative rat. Arrowheads indicate stimulus pairing sessions followed by infusion of CCK (red) or ACSF (blue). ***E***, decision index of individual animals (hollow circle) and grouped data (mean ± SEM) before and after stimulus pairings. *n* = 6, *p < 0.05, paired *t* tests.

To determine whether the local application of CCK enables the formation of the visuoauditory association in the AC of the anesthetized rat, we followed our previous paradigm (Chen et al., 2013) and used EACs as cues in the two-choice task. The rat was initially trained to detect the EAC in the left or right hemisphere and approach to the right or left hole respectively, for water reward (Fig. 1*A*). Once the rat reached a success rate of 90% for both the left and right EAC, the rat was anesthetized, and after locally infusing CCK-8 into the AC via the implanted cannula the VS was then paired with the target side of EAC (Fig. 1*A*). During the test session, the VS was presented at a frequency of 1:100 relative to EACs, and the decision index was assigned to quantify the animal's behavior, in which “1” was assigned when the rat approached the target hole, “0” to the nontarget hole, and “0.5” not to any of the two holes, respectively. We deliberately chose the hole to which the rat approached with fewer chances as the target hole, showing a decision index of < 0.5 in the baseline test (see Fig. 1*B* for a representative subject and Fig. 1*C* for the group results; also see Movies 3 and 5). The rat preferably approached to the target side after the VS was paired with the EAC at the hemisphere contralateral to the target hole (see Fig. 1*B* for a representative subject and Fig. 1*C* for group data; decision index: 0.82 ± 0.06 at week 2 vs 0.40 ± 0.08 baseline, *p* = 3.29E-5; 0.93 ± 0.03 at week 3–4 vs 0.40 ± 0.08 baseline *p* = 2.41E-7; DF = 2, *F* = 25.02, *p* = 3.39E-7, one-way ANOVA with *post hoc* Tukey tests; Movies 4 and 6). The decision index at both week 2 and weeks 3–4 were significantly above the chance level 0.5 (baseline, *p* = 0.2614; week 2, *p* = 0.0002, week 3–4, *p* < 0.0001, compared with 0.5, single sample *t* test). Such an effect was not due to the nonspecific effect of the drug infusion since the rat still showed significant differences in its approach to the opposite side relative to the CCK-8 infusion site. Further evidence for this finding not being related to nonspecific drug effects was found when CCK-8 and ACSF were infused into their respective sides and VS was paired with the EAC of both sides during the same anesthetized session, The rats still showed significant preference to approach the target side (Fig. 1*D*,*E*, decision index from 0.15 ± 0.03 to 0.37 ± 0.05, *p*=0.0313, paired *t* test).

Movie 3. Behavioral response to VS before CCK pairing for subject #2. 10.1523/JNEUROSCI.1673-19.2019.video.3

Movie 4. Behavioral response to after CCK pairing for subject #2. 10.1523/JNEUROSCI.1673-19.2019.video.4

Movie 5. Behavioral response to VS before CCK pairing for subject #5. 10.1523/JNEUROSCI.1673-19.2019.video.5

Movie 6. Behavioral response to VS after CCK pairing for subject #5. 10.1523/JNEUROSCI.1673-19.2019.video.6

### The visuoauditory association established between the natural AS and the VS in the presence of CCK in the anesthetized rat

**Figure 2.**
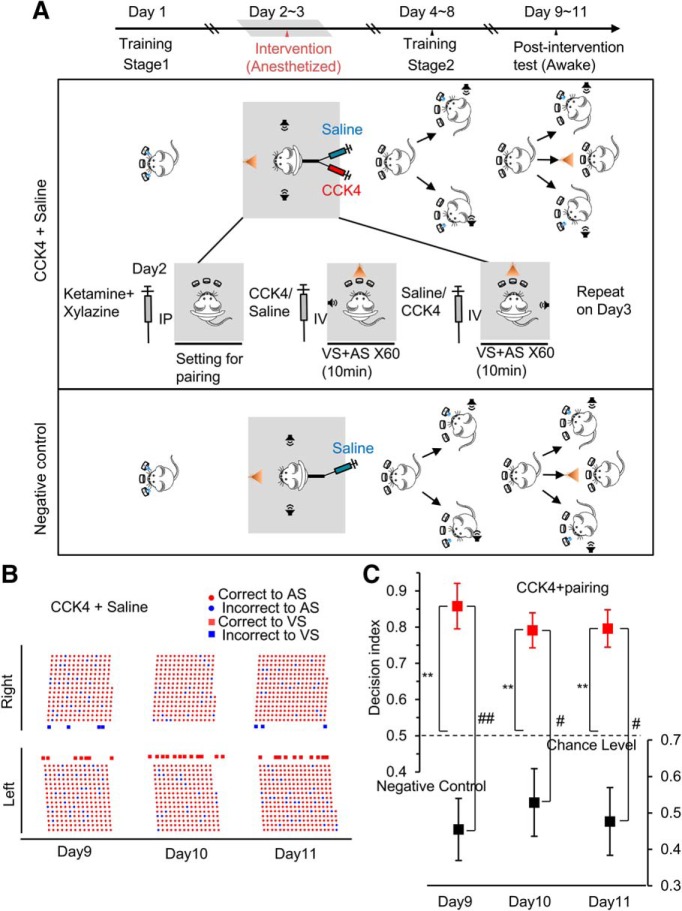
The visuoauditory association established between the VS and the AS in the presence of CCK-4. ***A***, Experiment timeline and schematic drawing of the experimental design for CCK-4 pairing and negative control groups. Gray shade represents the procedure under the anesthesia. ***B***, Behavioral performance during the postintervention test from day 9 to day 11. Raster display on the left shows the performance of an exemplary rat for its response to the AS in small dots and the VS in large black dots. The VS was paired to the AS on the left side when CCK-4 was administered. ***C***, Decision index for CCK-4 pairing and negative control groups over the 3 test days. Plots are in mean ± SEM. **p < 0.01, compared with chance level 0.5, single-sample *t* tests; #*p* < 0.05, ##*p* < 0.01, two-way ANOVA with pairwise comparisons.

Our next question was whether we could use the natural AS to replace the above EAC. We chose another CCKBR agonist, CCK-4, because studies have suggested that it is able to penetrate the blood–brain barrier (Bradwejn et al., 1990; Eser et al., 2009), which implied that we could apply it systemically through the tail vein without implanting infusion cannulas into the brain. Based on our earlier findings (Li et al., 2014; Chen et al., 2019), it is reasonable to assume that other than CCK application, the pairing of the inputs and neuronal activities are also important to induce neuroplasticity. The pairing of the VS and the AS at the target side was performed during CCK-4 application under anesthesia before the formal training (stage 2; Fig. 2*A*). In the anesthetized condition, rats (*n* = 7) were first subjected to systemic administration of CCK-4 and pairing of the VS and the AS of the target side, and then subjected to systemic injection of saline and pairing of VS and AS of the nontarget side (Fig. 2*A*). The rat was trained to retrieve water reward in the two-choice task, in which the left or the right AS guided the rats to approach corresponding holes (Fig. 2*A*). The rat took 5 d to reach a correct rate of 0.85 or above. The rat showed a significant preference to approach the target side across the three postintervention test days when the VS was randomly presented to the animal in a low incidence (1 of 30 trials; for a representative subject, see Fig. 2*B*; for the decision index of the group data, see Fig. 2*C*; decision index = 0.86 ± 0.06 on day 9, 0.79 ± 0.04 on day 10, 0.80 ± 0.05 on day 11; *p* = 4.2E-4, compared with chance level 0.5, single sample t tests). This preference was also significant compared with the negative control group where saline was injected before pairing the VS with the AS (Fig. 2*C*, bottom; two-way ANOVA, *F*_(2,26)_ = 1.374, *p* = 0.27; pairwise comparison, CCK group (*n* = 8) vs negative control group (*n* = 7), 0.86 ± 0.08 vs 0.46 ± 0.07 on day 9, *p* = 0.002, 0.79 ± 0.08 vs 0.53 ± 0.07 on day 10, *p* = 0.030, 0.80 ± 0.08 vs 0.48 ± 0.08 on day 11, *p* = 0.012). Such behavioral preference reflects that the VS was successfully associated with the AS of the target side under the influence of CCK.

### Responses of auditory neurons to the VS after pairing the VS and activation of the AC in the presence of CCK

**Figure 3.**
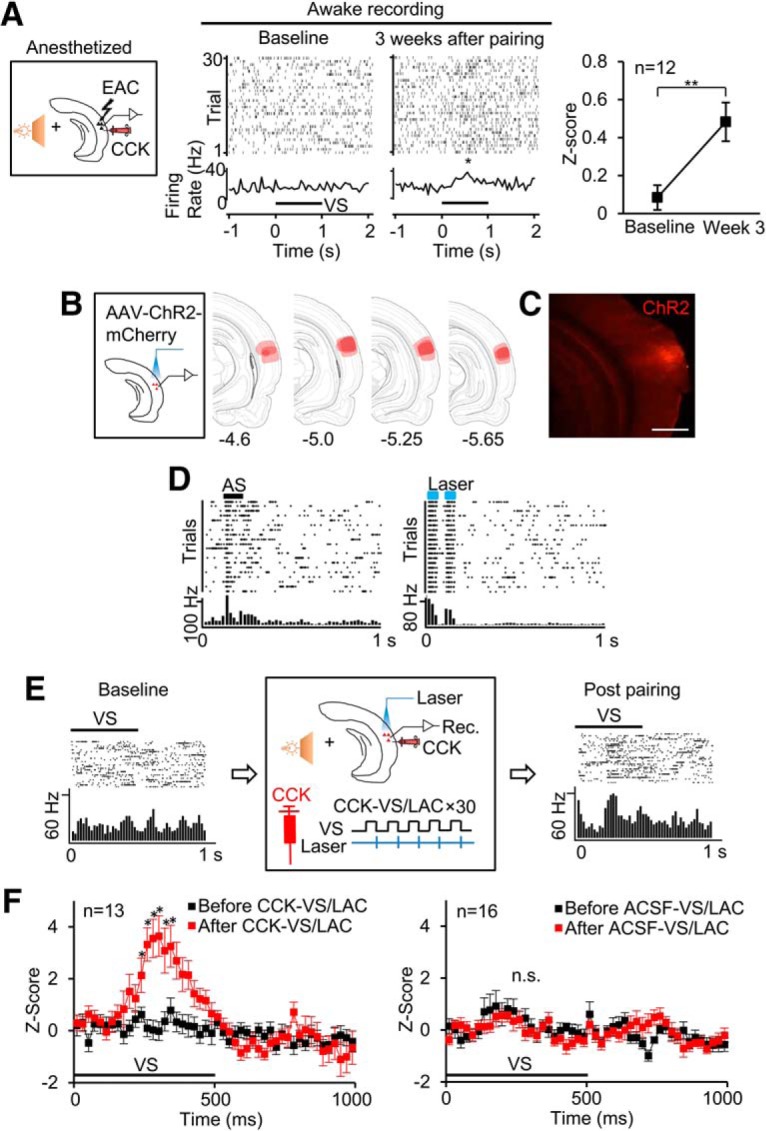
Changes of auditory cortical neuronal responses to the VS. ***A***, Neuronal responses to the VS before and after stimulus pairings. *Z*-scores were calculated based on differences between average neuronal firing during a 200 ms period after the VS and an equivalent period of spontaneous firing. Mean ± SEM, **p < 0.01, paired *t* tests. ***B***, Positions of virus injection in the AC and virus expression in the AC. The regions of viral expression (*n* = 3) were superimposed on redrawn coronal sections of rat brain atlas (Swanson, 2018). Numbers at the bottom are distance posterior to bregma (mm). ***C***, Representative image of ChR2 expression in AC. ***D***, Representative neuronal response to AS and laser in the AAV-CaMkIIa-ChR2-mCherry injection AC. ***E***, Raster plots and PSTHs show neuronal responses to the VS (light flash) before (leftmost) and after (rightmost) CCK infusion and stimulus pairings of VS/Laser stimulation of the AC (CCK-VS/LAC). The middle panel shows the procedure of the pairing. ***F***, *Z*-scores (mean ± SEM) show that the neuronal responses to the VS before and after the CCK-VS/LAC or the ACSF-VS/LAC. *Z*-scores were calculated based on differences between average neuronal firing on 20 ms time bins after the VS and an equivalent period of spontaneous firing. *p < 0.05, n.s., not significant, two-way ANOVA with Sidak's multiple-comparisons tests.

We next investigated whether neural plasticity occurred at the cellular level. We monitored the neural activity of the auditory neurons in response to the VS before and after the intervention of CCK-8s infusion and the pairing paradigm. In the awake rats, neurons in the AC, which did not respond to the VS before the intervention, responded to the VS after the intervention (Fig. 3*A*; baseline: 0.08 ± 0.07 vs week 3: 0.48 ± 0.10, *z*-score; *n* = 12; *p* = 2.06E-4, paired *t* test). We then questioned what type of neurons were involved in such neural plasticity and account for the visuoauditory association.

Given that excitatory cortical neurons are usually involved in neuroplasticity, we induced expression of the light-evoked channelrhodopsin-2 (ChR2) into the excitatory auditory neurons, and implanted an optic fiber and recording electrodes in the AC to stimulate and record these neurons (Fig. 3*B*,*C*). Neurons responded to the AS and laser stimulus (Fig. 3*D*). We investigated whether pairing the VS with the activation of these neurons in the presence of CCK-8 would change their response to the VS. Neuronal responses to the VS in the AC changed after paired presentation of the VS and laser stimulation of the AC (LAC) for 30 trials in the presence of CCK-8 (CCK-VS/LAC; Fig. 3*E*; population data in Fig. 3*F*, left; *Z*-score, DF = 1, *F* = 8.07, *p* = 0.009, two-way ANOVA), whereas no changes occurred after the same pairing preceded with ACSF infusion (ACSF-VS/LAC; Fig. 3*F*, right; *Z*-score, DF = 1, *F* = 0.07, *p* = 0.7883; two-way ANOVA). In the presence of CCK, pairing the VS and activation of the auditory neurons potentiated neuronal responses to the VS in the AC.

### HF stimulation of terminals of entorhinal CCK neurons in the AC enabled the potentiation of the neuronal response to the VS

**Figure 4.**
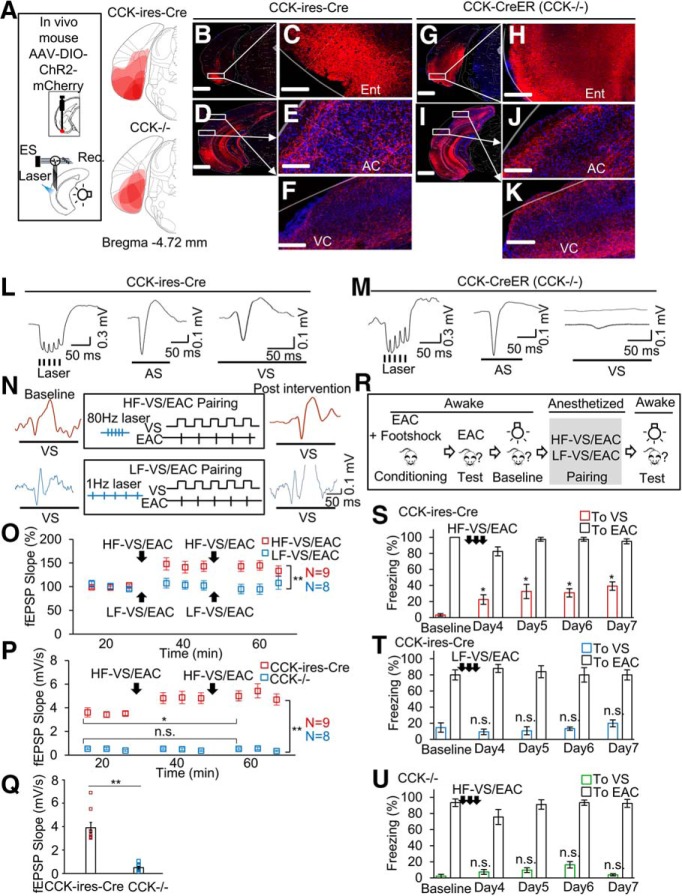
HF activation of CCK-containing entorhino-neocortical projections enables the association between the VS and EAC, leading to behavioral changes. ***A***, Schematic drawings for positions of virus injection in the entorhinal cortex and implantations of the laser fiber and stimulating/recording electrodes in the AC (AC). The regions of viral expression (*n* = 6 for CCK-ires-Cre, *n* = 4 for CCK^−/−^ mice) were superimposed on redrawn coronal sections of mouse brain atlas (Paxinos and Franklin, 2001). ***B***, AAV-DIO-ChR2-mCherry was injected into the entorhinal cortex of CCK-ires-Cre mice. Images show virus expression in the entorhinal cortex (***B–C***), the AC (***D–E***), and the visual cortex (VC) (***D***, ***F***). ***G***, AAV-DIO-ChR2-mCherry was injected into the entorhinal cortex CCK-CreER (CCK^−/−^) mice. Images show virus expression in the entorhinal cortex (***G–H***), the AC (***I–J***), and the VC (***I***, ***K***). Scale bars: 500 μm for ***B***, ***D***, ***G***, ***I***; 100 μm for ***C***, ***E***, ***F***, ***H***, ***J***, and ***K***. ***L***, fEPSPs to laser pulse train in the AC, AS, and VS of CCK-ires-Cre mice. ***M***, fEPSPs to laser pulse train in the AC, AS, and VS of CCK^−/−^ mice. ***N***, Representative fEPSPs before (left) and after (right) the HF-VS/EAC and LF-VS/EAC protocols (middle). The top shows fEPSPs and the protocol of HF-VS/EAC, and the bottom shows fEPSPs and the protocol of LF-VS/EAC. ***O***, Normalized slopes of fEPSPs after the HF-VS/EAC (red) or LF-VS/EAC (blue) pairing in CCK-ires-Cre mice. **p < 0.001, two-way ANOVA. ***P***, Slopes of fEPSPs (mean ± SEM) after the HF-VS/EAC (red) or LF-VS/EAC (blue) pairing in CCK^−/−^ mice. ***Q***, baseline fEPSP slopes (mean ± SEM plus individual values) to VS in CCK-ires-cre and CCK^−/−^ mice. ***R***, Cued fear conditioning and stimulation protocols. ***S***–***U***, Bar charts show the percentage (mean ± SEM) of time spent freezing in response to the conditioned EAC and the paired VS before and after the HF-VS/EAC (***S***) or LF-VS/EAC (***T***) pairing in CCK-ires-Cre mice, and HF-VS/EAC pairing in CCK^−/−^ mice (***U***). *p < 0.05, n.s., not significant, one-way ANOVA.

Our recent study showed that HF stimulation of the entorhinocortical terminals enabled the formation of a sound–sound association (Chen et al., 2019). The next question is whether the same projection enables the formation of the crossmodal visuoauditory association. To verify our hypotheses that HF stimulation is sufficient to trigger entorhinal CCK neurons to release CCK at their terminals in the AC where after the released CCK enables auditory neurons to respond to the VS after the VS/EAC pairing, we induced ChR2 expression in entorhinal neurons driven by Cre recombinase in CCK-ires-Cre and CCK-CreER (CCK^−/−^) mice. We implanted optic fiber and electrode arrays in the AC for both stimulation and recording (Fig. 4*A*). CCK neurons expressing ChR2 were labeled in the entorhinal cortex of both mice (Fig. 4*B*,*C* and *G*,*H*). Their terminals were found in both auditory (Fig. 4*D*,*E* and *I*,*J*) and visual cortices (Fig. 4*D*,*F* and *I*,*K*). Field EPSPs (fEPSPs) evoked by laser stimulation (80 Hz pulse train) toward terminals in the AC was found in both CCK-ires-Cre (Fig. 4*L*, left) and CCK^−/−^ mice (Fig. 4*M*, left), indicating that entorhinal CCK neurons that project to the AC were mainly excitatory. Neurons in the AC of both mouse types showed responses to the AS, as shown by the fEPSPs (Fig. 4*L*,*M*, middle). Moreover, we observed fEPSP responses to the VS in the AC of CCK-ires-Cre mice (Fig. 4*L*, right), but little to no response in the AC of CCK^−/−^ mice (Fig. 4*M*, right). The weak visual response in the AC of the CCK^−/−^ mice might reflect a deficit in the cross-modal association stemming from a lack of CCK.

Next, we adopted a pairing paradigm (Fig. 4*N*) in which HF laser activation of entorhinocortical CCK terminals in the AC was followed by five paired presentations of the VS and EAC. The pairing paradigm (referred to as HF-VS/EAC) was repeated four times. We used the same mouse type and a similar paradigm but with LF laser activation (LF-VS/EAC) of entorhinal CCK terminals in the AC as the control (Fig. 4*N*). The neuronal responses to the VS (fEPSP slope) increased after the HF-VS/EAC (Fig. 4*O*, two-way ANOVA, *F*_(1,15)_ = 17.631, *p* = 0.001 significant interaction; pairwise comparison, 96.0 ± 5.4% baseline vs 152.1 ± 8.7% after HF pairing, *p* = 1.17E-4, *n* = 9). There were no changes in the fEPSP slope after the LF-VS/EAC pairing (Fig. 4*O*, two-way ANOVA, *F*_(1,15)_ = 17.631, *p* = 0.001 significant interaction; pairwise comparison, 96.4 ± 9.2% baseline vs 106.9 ± 5.7% after LF pairing, *p* = 0.378, *n* = 8). There was a significant difference in the changes of fEPSPs between the experimental and control groups at the end of the recording (Fig. 4*O*, two-way ANOVA, *F*_(1,15)_ = 17.631, *p* = 0.001 significant interaction; pairwise comparison, 106.9 ± 5.7% after LF pairing vs 152.1 ± 8.7% after HF pairing, *p* = 0.001), indicating that the potentiation in the neuronal responses to the VS could be induced with HF-VS/EAC, but not with LF-VS/EAC.

In contrast, no enhancement in the neuronal response to the VS was observed in the AC of CCK^−/−^ mice with the same manipulation (Fig. 4*P*, two-way ANOVA *F*_(1,15)_ = 5.767, *p* = 0.0297 significant interaction; pairwise comparison, after pairing, CCK-ires-Cre (*n* = 9) vs CCK^−/−^ (*n* = 8), 5.6 ± 0.41 vs 0.43 ± 0.44, *p* < 0.001; pairwise comparison, CCK-ires-Cre before vs after, 3.9 ± 0.34 vs 5.6 ± 0.41, *p* < 0.001; pairwise comparison, CCK^−/−^, before vs after, 0.51 ± 0.36 vs 0.43 ± 0.44, *p* = 0.885) suggesting that CCK is a key molecule responsible for such HF-induced LFP enhancement. The fEPSP to the VS in the AC was detectable only from 3 of 8 CCK^−/−^ mice (for examples, see Fig. 4*M*, right). However, there was a huge difference in the slope of fEPSP to the VS in the AC between the CCK-ires-Cre and CCK^−/−^ mice even before the pairing (Fig. 4*Q*; 3.9 ± 0.34 vs 0.51 ± 0.36, *p* < 0.001, *t* test), indicating a possible deficit in the cortical associative connectivity between different modalities in the CCK^−/−^ mice.

### HF stimulation-induced LFP during anesthesia drove behavioral changes

We hypothesized that the visuoauditory association represented by an enhanced neuronal response to the VS during the above paradigm in anesthetized mice should be reflected in behavioral experiments, so we preconditioned the EAC of mice with a foot shock for 3 d before pairings and then compared the behavioral responses to the VS after the pairing with that occurring before the pairing (Fig. 4*R*). The CCK-ires-Cre mice showed significantly increased freezing responses to the VS after HF-VS/EAC (Fig. 4*S*; two-way ANOVA, *F*_(4,56)_ = 6.2, *p* = 3.4E-4 significant interaction; pairwise comparison, 3.33 ± 1.26% baseline vs 22.5 ± 5.84% at day 4, *p* = 0.040, *n* = 8; Movies 7, 8, 9, 10), but not after LF-VS/EAC (Fig. 4*T*; two-way ANOVA, *F*_(4,32)_ = 0.91, *p* = 0.468; pairwise comparison, 14.7 ± 6.04% baseline vs 9.3 ± 4.22% at day 4, *p* = 1.000, *n* = 5; Movies 11, 12, 13, 14). The fear response to the VS was maintained at day 7, or 4 d after the last HF-VS/EAC pairing (Fig. 4*S*; two-way ANOVA, *F*_(4,56)_ = 6.2, *p* = 3.4E-4 significant interaction; pairwise comparison, 3.33 ± 1.26% baseline vs 39.2 ± 4.53% at day 7, *p* = 0.034, *n* = 8). The CCK^−/−^ mice, however, showed no increase in their freezing responses to the VS after HF-VS/EAC pairing (Fig. 4*U*; two-way ANOVA, *F*_(4,64)_ = 2.073, *p* = 0.095; pairwise comparison, 2.22 ± 3.69% baseline vs 7.41 ± 6.91% at day 4, *p* = 1.000, *n* = 9).

Movie 7. Behavioral response to VS before EAC training for animal #3. 10.1523/JNEUROSCI.1673-19.2019.video.7

Movie 8. Behavioral response to VS after HF-VS/EAC pairing for animal #3. 10.1523/JNEUROSCI.1673-19.2019.video.8

Movie 9. Behavioral response to VS before EAC training for animal #3. 10.1523/JNEUROSCI.1673-19.2019.video.9

Movie 10. Behavioral response to VS after EAC training for animal #3. 10.1523/JNEUROSCI.1673-19.2019.video.10

Movie 11. Behavioral response to VS before LF-VS/EAC pairing for animal #2. 10.1523/JNEUROSCI.1673-19.2019.video.11

Movie 12. Behavioral response to VS after LF-VS/EAC pairing for animal #2. 10.1523/JNEUROSCI.1673-19.2019.video.12

Movie 13. Behavioral response to VS before EAC training for animal #2. 10.1523/JNEUROSCI.1673-19.2019.video.13

Movie 14. Behavioral response to VS after EAC training for animal #2. 10.1523/JNEUROSCI.1673-19.2019.video.14

### Visuoauditory direct projections strengthened after HF activation of entorhino-neocortical fibers

**Figure 5.**
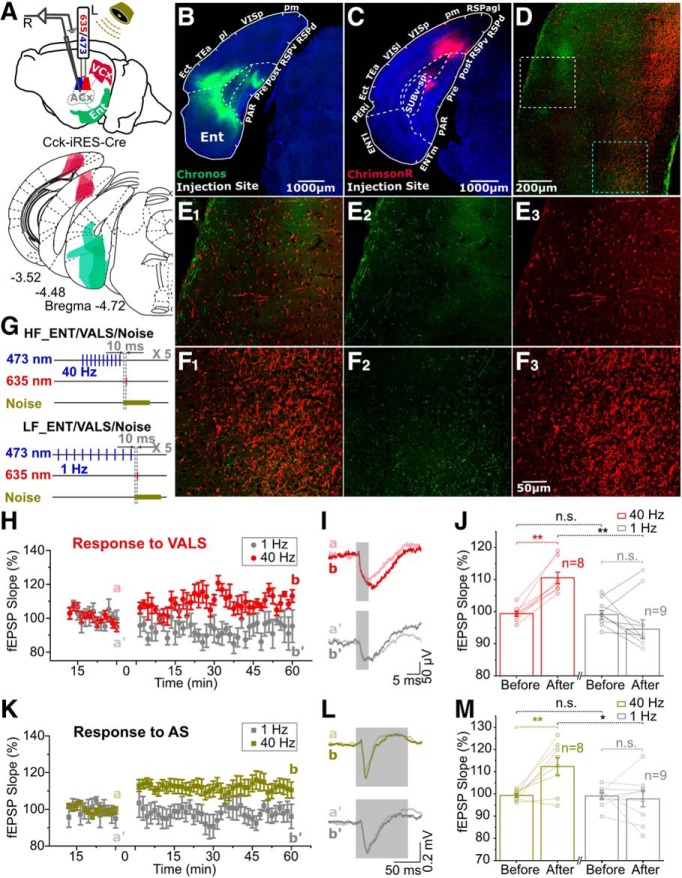
Visuo-auditory direct corticocortical projection strengthened after HF activation of entorhino-neocortical CCK^+^ fibers. ***A***, Diagram of the experiment design; 473 nm and 635 nm lasers were used to activate the projecting terminals with opsins expression in the AC from entorhinal and visual cortex of Cck-ires-Cre mice, which were transfected by AAV9-syn-Flex-Chronos-GFP (green) and AAV9-syn-ChrimsonR-tdTomato (red) respectively. Glass pipette electrodes were used to record the field EPSPs evoked by natural sound stimuli and different laser stimulations. The regions of viral expression (*n* = 3) were superimposed on redrawn coronal sections of mouse brain atlas (Paxinos and Franklin, 2001). ***B***, Chronos-GFP (green) expression at the injection site in entorhinal cortex. Blue is DAPI Staining. ***C***, ChrimsonR-tdTomato (red) expression at the injection site in visual cortex. ***D***, Projections from entorhinal CCK^+^ neurons (green) and visual cortical universal neurons (red) could be found in both superficial layers (white dashed rectangle) and deep layers (blue dashed rectangle) of AC, which are enlarged in ***E*** and ***F*** respectively. ***E1*** is merged by ***E2*** and ***E3***, and ***F1*** is merged by ***F2*** and ***F3***. Scale bars: 1000 μm for ***B*** and ***C***, 200 μm for ***D***, and 50 μm for ***E*** and ***F***. ***G***, Protocols of using HF (40 Hz, top) or LF (1 Hz, bottom) laser stimulation (473 nm) on entorhinal CCK^+^ projecting terminals (HF_Ent or LF_Ent) followed by pairing of laser stimulation (635 nm) on visuoauditory projecting terminals (VALS) and natural sound stimulus (noise). ***H***, Normalized slopes of fEPSPs evoked by VALS before *versus* after HF_Ent/VALS/noise (red circles) or LF_Ent/VALS/noise (gray circles) pairing protocol. ***I***, Representative single fEPSP_VALS_ trace before versus after HF_Ent/VALS/noise (a vs b, top) or LF_Ent/VALS/noise (a′ vs b′, bottom) pairing protocol. Gray transparent rectangles indicate the laser stimulations. (Scale bar: 5 ms and 50 μV.) ***J***, Statistics for normalized slopes of fEPSP_VALS_ (two-way RM ANOVA, significant interaction *F*_(1,15)_ = 21.326, *p* = 0.00033; pairwise comparison, increased by 11.2 ± 2.2%, before vs after HF_Ent/VALS/noise, *p* = 0.00017, *n* = 8; pairwise comparison, changed by 3.1 ± 2.1%, before vs after LF_Ent/VALS/noise, *p* = 0.16253, *n* = 9; pairwise comparison, 96.6 ± 2.4% vs 110.5 ± 2.5%, after LF_Ent/VALS/noise vs after HF_Ent/VALS/noise, *p* = 0.00121, *n* = 9 (1 Hz), 8 (40 Hz); pairwise comparison, 99.7 ± 1.1% vs 99.4 ± 1.2%, before LF_Ent/VALS/noise vs before HF_Ent/VALS/noise, *p* = 0.83437, *n* = 9 (1 Hz), 8 (40 Hz).) All data are mean ± SEM *p < 0.05; **p < 0.01; n.s., no significance. ***K***, Normalized slopes of fEPSPs evoked by noise before *versus* after HF_Ent/VALS/noise (olive squares) or LF_Ent/VALS/noise (gray squares) pairing protocol. ***L***, Representative single fEPSP_noise_ trace before versus after HF_Ent/VALS/noise (a vs b, top) or LF_Ent/VALS/noise (a' vs b', bottom panel) pairing protocol. Gray transparent rectangles indicate the noise stimuli. (Scale bar: 50 ms and 0.2 mV.) ***M***, Statistics for normalized slopes of fEPSP_noise_ (two-way RM ANOVA, significant interaction *F*_(1,15)_ = 8.296, *p* = 0.01144; pairwise comparison, increased by 13.1 ± 3.6%, before vs after HF_Ent/VALS/noise, *p* = 0.00260, *n* = 8; pairwise comparison, changed by 1.3 ± 3.4%, before vs after LF_Ent/VALS/noise, *p* = 0.71249, *n* = 9; pairwise comparison, 97.8 ± 3.7% vs 112.4 ± 3.9%, after LF_Ent/VALS/noise vs after HF_Ent/VALS/noise, *p* = 0.01578, *n* = 9 (1 Hz), 8 (40 Hz); 99.1 ± 1.2% vs 99.3 ± 1.3%, before LF_Ent/VALS/noise vs before HF_Ent/VALS/noise, pairwise comparison, *p* = 0.88270, *n* = 9 (1 Hz), 8 (40 Hz)). All data are means ± SEM *p < 0.05; **p < 0.01; n.s., no significance.

The above experiment demonstrated that HF activation of entorhino-neocortical fibers if followed by the pairing of the VS and electrical stimulation of the AC, could potentiate a marked neuronal response to the VS. We further investigated whether the convergence of entorhinal and visual projections in the AC accounts for this visuoauditory association. We injected two viruses into the entorhinal cortex (AAV-syn-flex-Chronos-GFP) and the visual cortex (AAV-syn-ChrimsonR-tdTomato) of the CCK-ires-Cre mice (Fig. 5*A*). The virus in the entorhinal cortex selectively transfected the CCK neurons (Fig. 5*B*), while the virus in the visual cortex transfected neurons in the visual cortex nonspecifically (Fig. 5*C*). Virus-labeled projections from both the visual cortex and the entorhinal cortex were found in superficial layers (Fig. 5*D*,*E*) and deep layers (Fig. 5*D–F*) of the AC.

To verify that HF stimulation (HFS) but not LF stimulation (LFS) of the entorhino-cortical projection terminals in the AC enables neuronal plasticity locally, we designed a pairing paradigm, as shown in Figure 5*G*, in which either HF (40 Hz) or LF (1 Hz) blue light stimulation was followed by a pair of red-light stimuli in the AC and a natural sound stimulus. Five pairings after the HFS on the terminals of entorhinal CCK neurons induced LTP of fEPSP to laser stimulation of the visuoauditory terminals, while that after LFS did not (LTP curves, Fig. 5*H*; fEPSP traces, Fig. 5*I*; two-way RM ANOVA, *F*_(1,15)_ = 21.326, significant interaction *p* = 3.3E-4; pairwise comparison, increased by 11.2 ± 2.2%, *p* = 1.7E-4, *n* = 8, after vs before HFS; pairwise comparison, changed by 3.1 ± 2.1%, *p* = 0.16, *n* = 9, after vs before LFS; pairwise comparison, after pairing: 110.5 ± 2.5% HFS vs 96.6 ± 2.4% LFS, *p* = 1.2E-3; Fig. 5*J*).

The neuronal responses represented by the fEPSP to the natural auditory stimulus was also examined after the above HF and LF protocols. We found that fEPSPs to the noise stimulus were significantly potentiated after the HFS but not the LFS (LTP curves, Fig. 5*K*; fEPSP traces, Fig. 5*L*; two-way RM ANOVA, *F*_(1,15)_ = 8.296, significant interaction *p* = 0.011; pairwise comparison, increased by 13.1 ± 3.6%, *p* = 0.0026, *n* = 8, after vs before HFS; pairwise comparison, changed by 1.3 ± 3.4%, *p* = 0.71, *n* = 9, after vs before LFS; pairwise comparison, 112.4 ± 3.9% HFS vs 97.8 ± 3.7% LFS, *p* = 0.016; Fig. 5*M*).

These results indicate that pairing a visual input to the AC in conjunction with a natural sound stimulus, induced LTP of the visuoauditory direct projections when HFS of the entorhinal projections was applied. This suggests that HFS of CCK positive projections triggers the release of CCK in the AC.

## Discussion

Infusion of CCK into the AC of anesthetized rats produced a neuroplastic change that enabled auditory neurons to start responding to a VS after the VS was paired with EAC. This visuoauditory association lasted for >3 weeks. Not only did AC neurons respond to the VS in awake rats, but this association between the VS and activation of the AC formed under anesthesia was translated into noticeable behavioral changes. In the presence of CCK, the visuoauditory association was also established between the VS and the natural AS and was additionally reflected in a similar behavioral task.

Pairing the VS and the stimulation of the excitatory neurons in the AC in the presence of CCK changed the neuronal response to the VS. HF activation of the terminals of the entorhinal CCK neurons in the AC, followed by pairing the VS and EAC, enhanced neuronal responses to the VS in the AC of anesthetized mice. When the mice were preconditioned to associate a foot shock with EAC, followed by pairing EAC with a VS, they showed significantly increased freezing responses to the VS after the pairings. The fear response to the VS was maintained for 4 d after the HF-VS/ES pairing. Neither potentiation in neuronal response to the VS, nor freezing response to the VS was observed from the CCK^−/−^ mice after the same operation. Similarly, after HF activation of the terminals of entorhinal CCK neurons in the AC, pairing laser stimulation of the projections from the visual cortex in the AC with natural sound stimulus-induced LTP of neuronal responses to visuoauditory inputs and the natural sound stimulus.

In a previous study, we established an artificial association between a VS and EAC through classical conditioning, such that AC neurons began responding to a VS (Chen et al., 2013). Inactivation of the entorhinal cortex blocked the establishment of this association. Previous experiments in rats demonstrated that inactivation of the perirhinal cortex disrupts the encoding, retrieval, and consolidation of object recognition memory (Winters and Bussey, 2005), whereas deep-brain stimulation of the medial temporal cortex in humans boosts memory performance (Suthana et al., 2012). Together, these findings add to our current understanding that the formation of associative memories requires interactions between the neocortex and the hippocampal system (Scoville and Milner, 1957; Corkin, 1984).

CCK is the most abundant of all neuropeptides (Rehfeld, 1978), and heavy labeling of CCK-containing neurons is observed in the entorhinal cortices (Innis et al., 1979). Recently, we found that retrogradely labeled neurons in the entorhinal cortex after the infusion of a tracer in the AC are mostly CCK-containing neurons. Moreover, activation of the entorhinal cortex enabled neuronal plasticity in the AC, but this was minimized by local infusion of CCK antagonists into the AC (Li et al., 2014; Chen et al., 2019). Therefore, we propose that the hippocampal system sends a memory-encoding signal, the neuromodulator CCK, to the neocortex to enable the formation of a new associative memory through its entorhinal gateway. Aside from the functional marriage of the hippocampal and entorhinal systems, CCK also facilitates neuronal plasticity in hippocampal neurons *in vitro* (Deng et al., 2010).

Further support for our hypothesis that CCK plays a critical role in behavior, are studies showing that blockade of CCK receptors suppresses conditioned fear (Tsutsumi et al., 1999), and deletion of the CCKB receptor gene reduces anxiety-like behavior (Horinouchi et al., 2004). Furthermore, activation of CCKB receptors in the amygdala potentiates the acoustic startle response (Fendt et al., 1995; Frankland et al., 1997), and application of a CCKB receptor antagonist attenuates fear-potentiated startle (Josselyn et al., 1995). Findings that mice lacking the CCK gene exhibit poor performance in a passive avoidance task and impaired spatial memory (Lo et al., 2008) could be interpreted as the result of a memory-encoding deficit in the brain.

People have been trying to induce memories under anesthesia for decades. For example, an early study demonstrated Pavlovian fear conditioning induced by epinephrine under anesthesia in rats (Weinberger et al., 1984). More recently, using fear conditioning and optogenetic techniques in rats, memory-encoding neurons created a false association between a particular context and a foot shock that was never delivered (Ramirez et al., 2013). Similarly, we implanted an artificial association between a VS and an EAC stimulus upon CCK infusion while rats were under anesthesia. Subsequently, the rats used an existing association between the EAC and a water reward and thus used the VS to guide their retrieval of the reward. More specifically, we demonstrated a hippocampal-cortical circuit underlying such plasticity. Although the visuoauditory associative memory implanted in the AC was quickly reflected in neuronal responses to the VS, it took several days before the association was reflected in the rats' behavior. This delay may be due to the time needed for the newly implanted memory in the neocortex to be registered in the hippocampus (Teyler and DiScenna, 1986) or to be linked with place cells in the hippocampus for directional guidance (O'Keefe and Dostrovsky, 1971).

It has been reported that certain types of emotional associative learning occur even when subjects are anesthetized, and such associative memory is hippocampus-independent (Rosenkranz and Grace, 2002). In our control experiment, where the VS and the AS were paired after saline injection, animals showed no behavioral changes in response to the VS that went beyond the level of chance. No detectable changes in the visual responses in the AC were observed after repetitive pairing of the VS and the EAC with ACSF infusion or LF stimulation of CCK terminals at the cellular level. These data suggest that the general visuoauditory association by simply pairing visual and auditory stimulation under anesthesia may occur at a minimum level that is not sufficient to be detectable in our case. Other components, such as aversive stimulation might be necessary for learning during anesthesia, which is not involved in our intervention.

Deep-brain stimulation of the entorhinal cortex, while subjects learned locations of landmarks enhanced their subsequent memory of these locations (Suthana et al., 2012). Evidence suggests that coordinated oscillation of entorhinal and hippocampal neurons in 20–40 Hz links to the encoding and retrieval of the associative memory (Igarashi et al., 2014), whereas gamma-band (40–120 Hz) oscillation links to cortical plasticity and formation of new memories (Osipova et al., 2006; Headley and Weinberger, 2011). However, multimodal associative memory is likely based on the association of multiple cortical areas (Andersen, 1997; Calvert et al., 1997). Formation of paired visual associations or paired visuoauditory associations critically depends on the perirhinal and entorhinal cortex (Higuchi and Miyashita, 1996; Chen et al., 2013). In the present study, we showed that HF activation, but not LF stimulation, of the neocortical projection terminals of entorhinal neurons, leads to LTP in fEPSPs to the VS in the AC. Modifying synaptic strength leads to memory formation (Nabavi et al., 2014). The potentiated neuronal responses to the VS led to an increased freezing time when the VS was presented, indicating that an association between the AS and ES in the AC was strengthened.

Given that we used CCK-ires-Cre mice, neurons infected with AAV-DIO-ChR2-mCherry in the entorhinal cortex were CCK neurons. Earlier studies indicate that the release of neuropeptides occurs slowly in response to repetitive firing (Whim and Lloyd, 1989; Shakiryanova et al., 2005). Our recent study showed that NMDA receptors control the release of CCK from entorhinocortical projection in the neocortex, and the released CCK enables cortical LTP and sound–sound associative memory (Chen et al., 2019).

Consistent with previous findings that CCK is associated with learning and memory (Josselyn et al., 1995; Tsutsumi et al., 1999; Chen et al., 2019), we found that CCK^−/−^ mice show deficits in associating the visual and auditory stimuli. CCK^−/−^ mice needed 1–2 times more conditioning trials to associate the sound cue with foot shock (even with a delayed conditioning protocol). In the present study, CCK-containing entorhinal neurons in CCK-ires-Cre mice labeled with AAV-DIO-ChR2-mCherry projected to the AC and visual cortex. Laser stimulation of these terminals elicited fEPSPs in the AC, suggesting that they are glutamatergic neurons. Association of artificial manipulation using electrical stimulation and a natural stimulus suggests potential neuroengineering and therapeutic applications. The rescuing effect of intravenous application of CCK-4 in forming associations between the VS and AS in the brain further proved that CCK is a key chemical for encoding visuoauditory associative memory in the brain.

These results, together with our three recent studies (Chen et al., 2013; Li et al., 2014; Chen et al., 2019), indicate that the entorhinal cortex is important for the establishment of visuoauditory associative memory and that CCK is involved in this bridging process. In the presence of CCK, neurons in the AC begin to respond to a VS after pairings of the VS and ES of the AC. Moreover, this altered neuronal response can be interpreted as an artificial memory in a behaviorally relevant context. Although we have more questions related to the gap in the temporal window between stimulus pairings and behavioral changes, as well as the processes that underpin extinguishing such associative memories, our study provides a new perspective on how memory is established in the neocortex. These results also provide insight for future efforts to rewire malfunctioning brains to bypass lost functions.
